# Virtual reality-based Harms tangent screen test for strabismus measurement

**DOI:** 10.1007/s00417-024-06724-2

**Published:** 2025-01-06

**Authors:** Yaroslava Wenner, Johann Schneider, Timo Drisser, Yu Yi Yang, Thilo Demeter, Maria Fronius, Birgit Lorenz, Thomas Kohnen, Michael Müller, Jochen Triesch

**Affiliations:** 1 Department of Ophthalmology, Goethe-University, Theodor-Stern-Kai 7, 60590 Frankfurt am Main, Germany; 2 Frankfurt Institute for Advanced Studies (FIAS), Frankfurt am Main, Germany; 3 TransMIT Centre of Translational Ophthalmology, c/o Justus-Liebig-University, Giessen, Germany

**Keywords:** Virtual reality, Harms tangent screen test, Strabismus, Torsion, Squint angle, Diplopia

## Abstract

**Purpose:**

Our study presents a virtual reality-based tangent screen test (VTS) to measure subjective ocular deviations including torsion in nine directions of gaze. The test was compared to the analogous Harms tangent screen test (HTS).

**Methods:**

We used an Oculus Go^®^ controller and head-mounted-display with rotation sensors to measure patient’s head orientation for the VTS. The software was developed with Unity^®^. We assessed subjective squint angles of adult patients with eye motility disorders of different origin in nine positions of gaze by means of HTS and VTS. We calculated mean difference, 95% limits of agreement and intraclass correlations (ICC) for horizontal, vertical and torsional deviations.

**Results:**

We included 85 patients. Measured horizontal and vertical deviations showed very good agreement between the two methods in all gaze directions (horizontal and vertical ICC: 0.93–0.98). Agreement was lower for torsional deviations (ICC: 0.79–0.93). The mean difference in primary position was 1.5° (95% limits of agreement -3.5° to 6.5°) for horizontal, 0° (-2,7° to 2,7°) for vertical, and 0.3° (-4.0° to 3.0°) for torsional deviations. The average examination time was 6 min with the VTS compared to 15 min with the HTS.

**Conclusions:**

Vertical and horizontal deviations showed good agreement between both tests measuring a slightly higher esodeviation with VTS probably due to an increased vergence demand. Measurement of torsional misalignment would benefit from a controller with more holding stability, such as a PC-mouse with central wheel, facilitating fine adjustments. VTS allowed automatic documentation, required less time and was easier to use than HTS.

****Key messages**:**

***What is known***

- The incidence of neurogenic diplopia increases in the ageing population.
- The Harms tangent screen test (HTS) reliably measures subjective ocular deviations in patients with diplopia.
- The HTS requires staff well-trained in orthoptics limiting its use.

***What is new***

- A novel virtual reality-based tangent screen test (VTS) enables measurement of horizontal, vertical and torsional ocular misalignment and shows a good comparability to the HTS.
- VTS uses commercially available head-mounted-display and controller, and specifically developed software, requires less time than HTS and allows automatic documentation.
- VTS administration does not require orthoptic training and can eventually be implemented in a clinical setting to provide a wider availability of subjective squint angle measurements.

**Supplementary Information:**

The online version contains supplementary material available at 10.1007/s00417-024-06724-2.

## Introduction

Timely examination of ocular deviations is essential for the diagnosis of eye movement disorders of different etiology, especially in case of neurogenic origin. A prism cover test allows objective measurement of the horizontal and vertical deviations but not of the torsional misalignment. One of the widely used methods to assess subjective squint angles in patients with diplopia is the Harms tangent screen test (HTS). It allows a detailed overview of the ocular misalignment and the eye muscles involved [1–3]. The binocular dissociative subjective test measures primary and secondary deviations including torsional misalignment in nine positions of gaze. Advantages of the method are low measurement error and precise definition of the viewing direction via the front projector with position cross [1–3]. Errors can occur due to an inaccurate alignment of the front projector or a wrong reading of the orientation. The HTS requires a space-consuming screen, is time consuming and needs specifically trained staff limiting its use.

VR technology enables a computer-generated representation of a three-dimensional (3D) environment. Each eye looks at a different image in the head-mounted-display (HMD) and the 3D impression is created by stereoscopic differences between the images. Head tracking enables the registration of head rotations using gyroscopic and accelerometer-based sensors. All these features make VR a well-suited technology for development of a digital HTS. The current use of VR technology is primarily limited to objective measurement of ocular deviations based on the size of the eye’s movement via eye tracking [4, 5]. Although good agreement of these tests with the alternating prism cover test (APCT) was reported, their main shortcoming is the lack of torsional misalignment data. Although digital versions of HTS requiring a beamer to display tangent screen have been recently developed [6, 7], we are not aware of any use of VR technology analogous to HTS.

We designed a novel VR-based tangent screen (VTS) for measurement of ocular deviations. The purpose of this study was to evaluate and compare VTS to HTS in patients with strabismus of various origins.

## Methods

### Inclusion and exclusion criteria

We conducted a prospective diagnostic study enrolling patients ≥ 18 years with concomitant, paralytic or restrictive strabismus causing diplopia seen at the Department of Ophthalmology, Goethe-University, Frankfurt am Main, Germany. Exclusion criteria were patients with visual acuity > 0.4 LogMAR, insufficient cooperation to perform HTS or VTS, eccentric fixation, anomalous retinal correspondence, or suppression not allowing subjective squint angle measurements. Further exclusion criteria were extreme angles of strabismus > 30 degrees and severe limitation above 15° of ocular ductions. We excluded patients with acute or chronic vertigo because VR technology can induce cybersickness [8]. We further excluded patients with epilepsy in order to avoid an epileptic seizure during the examination.

### Ophthalmological examination

Ocular deviations of patients with paralytic, restrictive or concomitant strabismus were assessed with a complete orthoptic examination including APCT at distance fixation (5 m) and at near (0.3 m). The patient's own glasses were worn during assessment of the ocular deviation in case of significant ametropia (< −1.0 dpt to > + 2.0 dpt). Additional examinations included medical history, measurement of objective refraction, best corrected visual acuity, examination of binocular vision, slit-lamp biomicroscopy of the anterior and posterior segment. Ocular ductions were assessed either by judging the corneal light reflex or by tangent screen examinations.

After verbal instructions to the patients HTS and VTS were performed at 2.5 m distance in nine diagnostic directions of gaze with sequential fixation of either eye. First, experienced orthoptists applied the HTS. Then, medical students applied the VTS.

### HTS

**Fig. 1:**
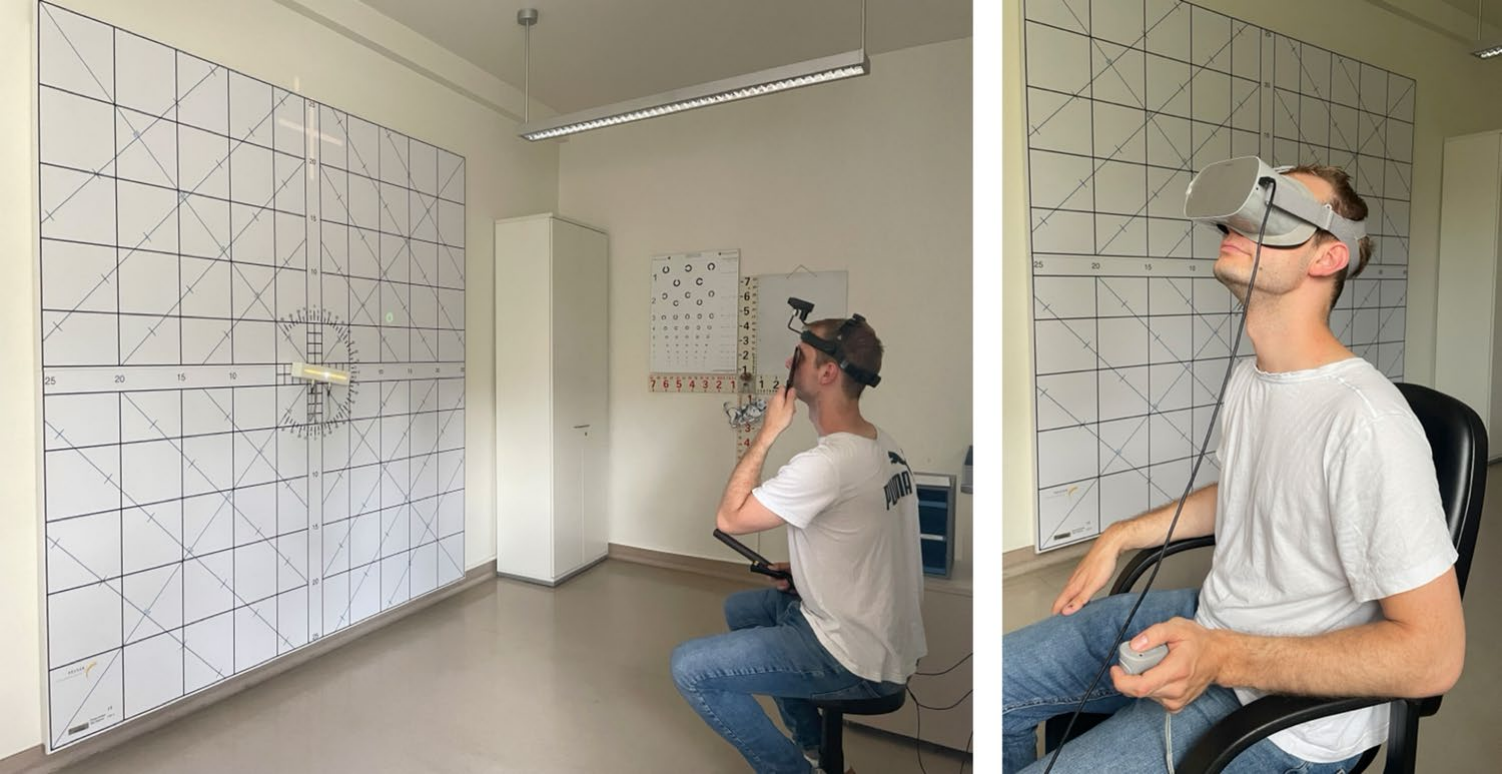
To the left: The Harms tangent screen with horizontal and vertical lines with degrees of graduation extending across the screen and the center bar with the fixation light. A patient wears a helmet with a forehead projector and holds a light pointer and a remote control. Left eye is covered by a dark red glass. To the right: The patient wears an Oculus Go^®^ Head-Mounted-Display and holds a controller to perform a virtual reality-based tangent screen test in 25° downgaze

A large standard HTS with boards dimension of 2.9 m in each direction was used (Fig. 1) [2, 3]. It displays a chessboard pattern with degrees of graduation extending across the screen. The fixation light is housed in the center of an oblong bar that can be converted to a fixation line for assessing cyclotorsion [2]. During the examination, the orientation of both eyes, the orientation of the head and the subjective visual impression of the patient were determined in nine directions of gaze including primary position (0°; 25°). A forehead projector mounted on the patient’s head projected a white cross on the board thus allowing the examiner to select the appropriate gaze direction. The fixating eye was covered by a dark red glass, which makes the fixation light appear red through the filter and does not allow any room localization. The patient indicated the perceived position on the screen using a large hand-held green light pointer and the degrees of horizontal and vertical deviations were noted. Since the two foveae are corresponding points, the red light and the green light of the pointer were perceived as superimposed [2]. Using the remote control, the patient rotated the illuminated bar to indicate the degree of perceived torsion. Once all measurements were recorded in each gaze position, the red filter was placed in front of the other eye and the test was repeated. The data were plotted on a form sheet with a grid to enter the horizontal, vertical, and torsional angles for each gaze position.

### VTS

**Fig. 2:**
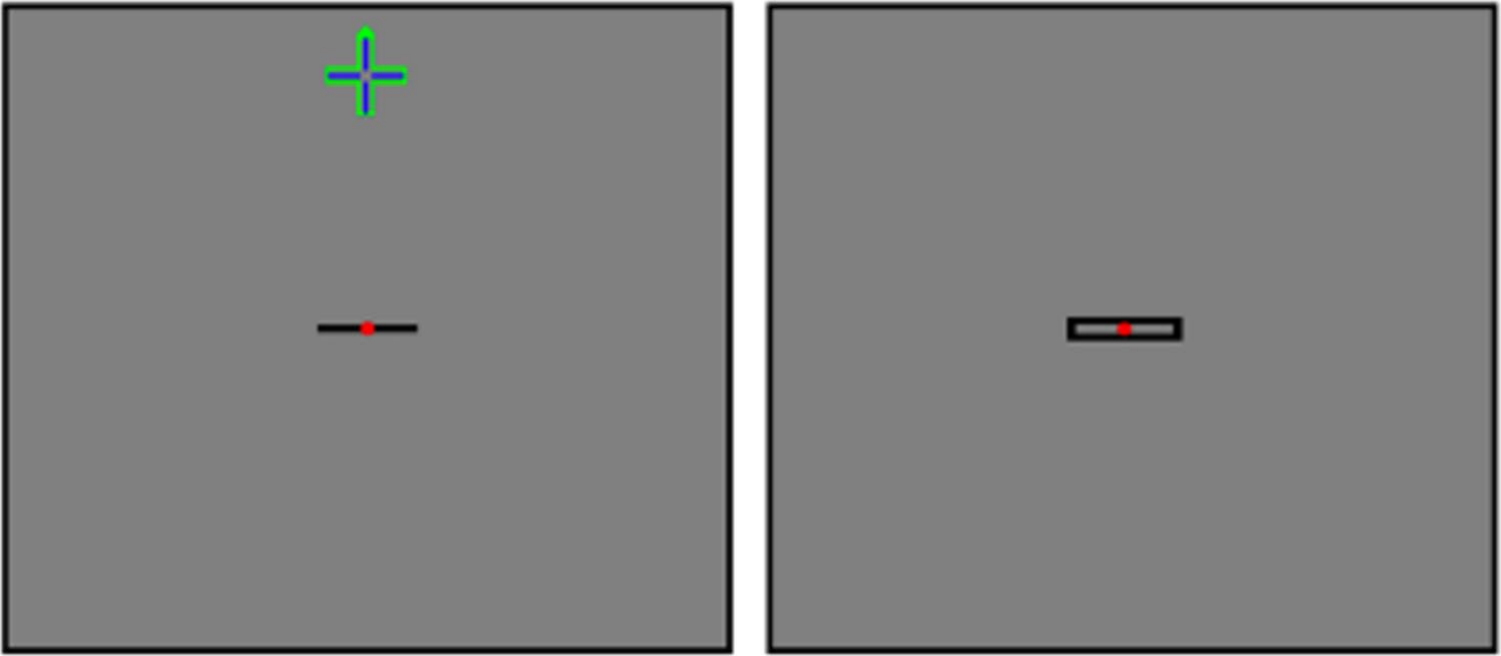
Virtual reality-based tangent screen test during runtime. The right eye is fixating and sees the right image. Left image is displayed to the left eye. The head is in up position indicated by green cross, so that angles of deviation are recorded in 25° downgaze

The software used comprises a number of vision measurement tests, including the VTS, and training games for visual disorders [9]. It was developed with Unity (Unity Technologies, USA) and runs on various commercially available HMDs. In our study a standalone HMD Oculus Go^®^ (Meta Reality Labs, USA) was used (Fig. 1). It provides a resolution of 1280 × 1440 pixels per eye with a refresh rate of 70 Hz, has a monocular field of view of 60° and a fixed interpupillary distance of 63.5 mm. The investigation was carried out at a virtual distance set to 2.5 m. Patients wore their glasses under the HMD as needed. When wearing an HMD each eye looks at a different part of the screen thus causing dissociation. The patient had to align a black line with a central red dot presented to the non-fixating eye exactly on the red dot and the frame presented to the fixating eye (Fig. 2). In case of detection of a tilt, the patient was asked to rotate the line with the right or left button on the top of the controller until the line appeared parallel to the frame. For the patient, red dots, the line and the frame appeared thereafter at identical positions. On the virtual screen, they were separated in case of strabismus. In analogy to the traditional HTS the process was repeated for all nine diagnostic directions of gaze first with the right eye fixating and then with the left eye fixating. At the end of the test, the horizontal, vertical and torsional deviations were calculated based on the position of the line and the frame on the screen.

**Fig. 3:**
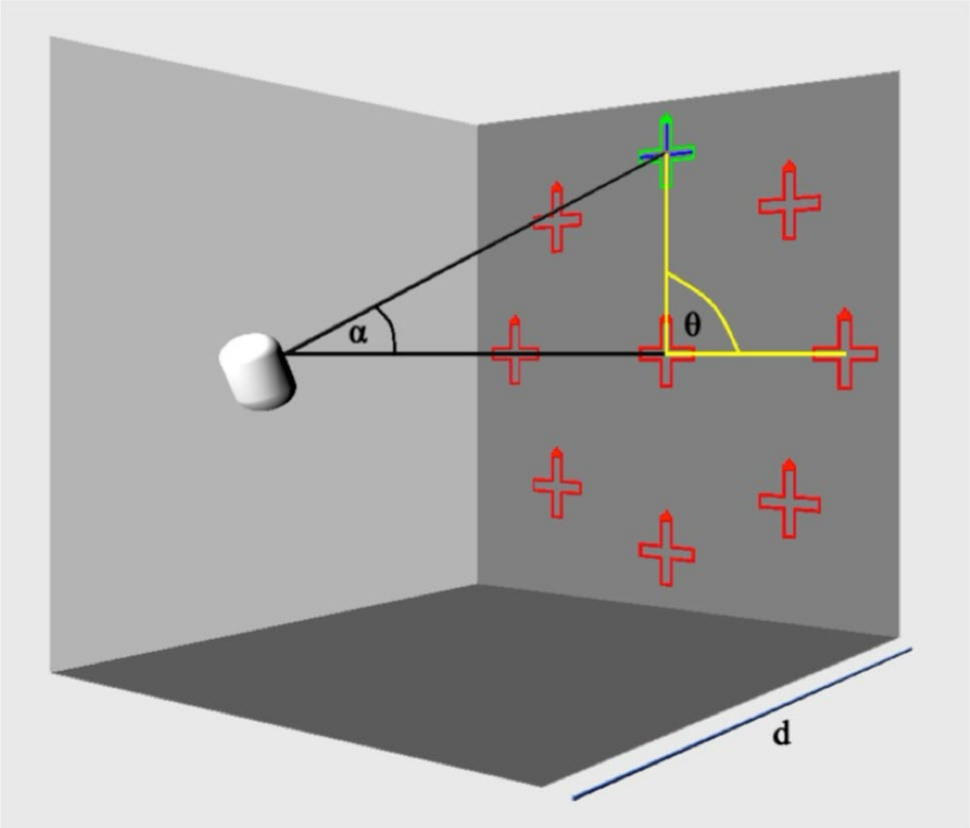
Schematic illustration of the virtual reality-based tangent screen test. The capsule represents the patient’s head and the red cross frames show the test positions at a test distance d. α represents the angle of the head rotation and θ is the test angle on the circular field of view

Head positions were calculated by the software using the following trigonometric equations (Fig. 3):

$$\mathrm{x }=\mathrm{ d }\cdot \mathrm{ tan}(\alpha ) \cdot \mathrm{ cos}(\theta )\text{ and y }=\mathrm{ d }\cdot \mathrm{ tan}(\alpha ) \cdot \mathrm{ sin}(\theta ),$$

where α was the angle between the primary and required head position. The different viewing directions were measured at α = 25°. In case of limited eye ductions the software allowed a manual reduction of the field of view in 5° steps separately for each gaze direction. θ corresponded to the angle on the circle of the planar representation of a sphere. When the head was turned to the right, this position was defined as θ = 0° and resulting viewing direction was 25° to the left. θ = 45° corresponded to a head rotation to the upper right and analogously the remaining viewing angles were calculated in 45° steps counterclockwise. Once the correct head position had been taken, the cross frame turned green (Fig. 2). Only in this case, the position of the bar in the frame could be confirmed.

We measured horizontal and vertical deviations in a polar coordinate system (elevation, azimuth, distance) that is projected onto the frontoparallel plane of the VTS. Horizontal (azimuth) and vertical (elevation) deviations were calculated using the following trigonometric equations:

$$\Delta\mathrm{x }= {\mathrm{tan}}^{-1} (\mathrm{x}/\mathrm{d})\text{ and }\Delta\mathrm{ y }= {\mathrm{tan}}^{-1} (\mathrm{y}/\mathrm{d}),$$

where d = 2.5 m was the test distance and x and y were the horizontal and the vertical deviation respectively of the bar to the frame on the tangent screen. Tilt angle between the bar and the frame corresponded to the degree of the cyclotorsion. For different coordinate systems to describe eye movements and squint angles see, e.g., Simonsz (1989) [10].

### Statistics

Results of squint angle measurements with the fixation of the non-paretic or non-deviating eye were used for statistical analysis. The differences between the measured squint angles were calculated:

$$\Delta\mathrm{Angle }=\text{ Angle VTS}-\text{ Angle HTS}.$$

The intraclass correlation coefficients (ICC) based on mean rating (k = 2), absolute agreement and two-way random effects were calculated to assess the agreement of the two tests. Following statistical formula was used [11]:

$$({\mathrm{MS}}_{\mathrm{R}}- {\mathrm{MS}}_{\mathrm{E}}) / ({\mathrm{MS}}_{\mathrm{R}}+ {\mathrm{MS}}_{\mathrm{C}}/{\mathrm{n}}- {\mathrm{MS}}_{\mathrm{E}}/{\mathrm{n}}),$$

in which MS_R_ = mean square for residual sources of variance; MS_E_ = mean square for error; MS_C_ = mean square for columns; n = number of subjects; k = number of measurements.

Additionally, absolute levels of agreement between the angles of deviation measured by VTS and HTS were calculated as a mean difference including the upper and lower limits of agreement (LOA) by determining 95% confidence interval. Differences between the angles of deviation with VTS and HTS were correlated with age, refraction, magnitude of the deviation and with type of eye motility disorder by means of multiple regression analysis with backward elimination. All analyses including the sample size calculation were performed using BiAS (© epsilon publishing 1989–2024 Dr. H. Ackermann, Goethe University Frankfurt). A difference in the measured squint angle of 2° was considered to be clinically relevant. Assuming a standard deviation of 3° and a maximal difference of 10° [12], this resulted in a case number of 70 eyes with significance level of 0.05 and a second-order error of 0.9.

## Results

The demographic characteristics of 85 patients included in the study are listed in Table 1. Fifteen patients were excluded for various reasons including low visual acuity (*n* = 2), difficulty to perform HTS (*n* = 1) or VTS (*n* = 4) due to their neurological condition, anomalous correspondence (*n* = 1), inappropriate refractive correction or glasses with prisms (*n* = 4), language barrier (*n* = 1) and absence of squint (*n* = 2). Most patients reached 25° viewing directions with VTS, but in 5 to 18 patients manual reduction of the field of view was necessary depending on gaze direction. Original data of each patient including strabological diagnosis, state of binocular vision, age, spheric equivalent and squint angles assessed with both methods in each gaze direction can be found in Table S1 in supplementary material.

**Table 1:** Demographic and clinical data of patients

Parameter	Patients (*n* = 85)
Age (year; mean, SD and range)	49.1 ± 17.4 (20–84)
SE (diopter; mean, SD and range)	−1.0 ± 2.3 (−11.5–3.0)
Amount of deviation by HTS (range)	
horizontal	−23° to 30°
Vertical	−22° to 18°
Torsional	6° in- to 17° ex
Type of strabismus (No)	
Esotropia	19
Exotropia	10
3rd nerve palsy	8
4th nerve palsy	25
6th nerve palsy	14
Restrictive strabismus	9

*SE* spherical equivalent, *HTS* Harms tangent screen test, *in* incyclotorsion, *ex* excyclotorsion

Results acquired with the VTS and HTS and 95% LOA were similar (Table 2). Horizontal and vertical deviations showed the best agreement (ICC: 0.93–0.98) in all viewing directions. The agreement was lower for torsional squint angles with ICC ranging from 0.79–0.93. VTS resulted in more measurements with 0° cyclodeviation (51.0%) compared to HTS (26.4%), which might have contributed to lower ICC.

**Table 2:** Intraclass correlation, mean difference and 95% limits of agreement for squint angles in different gaze directions between the VTS and HTS

*N* = 85*	Horizontal deviation			Vertical deviation			Torsional deviation		
Gaze direction	ICC	mean ± SD	LOA 95%	ICC	mean ± SD	LOA 95%	ICC	mean ± SD	LOA 95%
Primary position	0.97	1.5° ± 2.5°	−3.5°; 6.5°	0.98	0.01° ± 1.3°	−2.7°; 2.7°	0.93	−0.33° ± 1.8°	−4.0°; 3.3°
Right gaze	0.96	2.3° ± 2.4°	−2.5°; 7.0°	0.95	0.04° ± 2.0°	−4.0°; 3.9°	0.89	−0.32° ± 2.5°	−5.3°; 4.7°
Left gaze	0.96	2.1° ± 2.6°	−3.1°; 7.3°	0.98	−0.28° ± 1.6°	−3.5°; 2.9°	0.92	−0.63° ± 2.1°	−4.9°; 3.6°
Upgaze	0.96	2.2° ± 2.7°	−3.2°; 7.6°	0.97	0.11° ± 1.7°	−3.3°; 3.5°	0.92	0.13° ± 2.6°	−5.0°; 5.3°
Right upgaze	0.96	2.6° ± 2.4°	−2.2°; 7.4°	0.95	0.08° ± 2.1°	−4.2°; 4.3°	0.89	−0.71° ± 2.8°	−6.3°; 4.9°
Left upgaze	0.93	3.0° ± 3.2°	−3.5°; 9.4°	0.97	−0.35° ± 2.0°	−4.3°; 3.6°	0.93	−0.41° ± 2.7°	−5.0°; 4.1°
Downgaze	0.96	1.6° ± 2.8°	−4.1°; 7.3°	0.94	0.58° ± 2.1°	−3.5°; 4.7°	0.79	−0.53° ± 2.8°	−6.1°; 5.0°
Right downgaze	0.97	0.9° ± 2.6°	−4.3°; 6.2°	0.94	0.30° ± 2.1°	−3.9°; 4.5°	0.84	−0.67° ± 2.5°	−5.6°; 4.2°
Left downgaze	0.97	1.4° ± 2.4°	−3.4°; 6.2°	0.95	−0.24° ± 2.1°	−4.4°; 3.9°	0.87	−0.54° ± 2.1°	−4.7°; 3.7°

*in upgaze and right and left upgaze *N* = 84; ICC: intraclass correlation (k = 2) [11]; mean ± SD: mean difference and standard deviation between VTS and HTS; LOA 95%: 95% limits of agreement

*HTS* Harms tangent screen test, *VTS* virtual reality-based tangent screen

Horizontal squint angles showed on average higher esodeviation of 1.96° with the VTS compared to the HTS. The average difference of the vertical misalignment was 0.02° and of the torsional misalignment −0.54° respectively. Multiple regression analysis with backward elimination revealed no significant relationship between the amount of difference in squint angles between the two tests and the magnitude of the deviation, type of ocular motility disorder, refraction and age (*p* > 0.05).

Average acquisition time for both fixations with the VTS was 6.2±2.3 min, and with the HTS 15.0±4.1 min. None of the patients experienced nausea or dizziness during the VTS.

## Discussion

Traditional tests for mapping subjective ocular deviations such as the HTS, Lancaster red-green test, or the Hess screen test are established methods used for the diagnosis and follow-up of patients with strabismus [1–3]. However, all these tests require examiners experienced in orthoptics for error-free operating and recording of the data that limits their use and can lead to a diagnostic gap. Implementation of virtual reality technology in the assessment of ocular deviations could be a game changer for strabismus diagnostics. Our research group designed a novel VTS analogous to the HTS. The results of this test measuring horizontal, vertical and torsional deviations are encouraging. VTS runs with commercially available equipment, needs less time than HTS, has no measurement transmission errors and allows automatic documentation. Every technician can operate this easy-to-use technology. Currently, a disadvantage is the slightly higher handling difficulty especially for older patients that can probably be solved by using a conventional computer mouse interface.

Ocular deviations measured with the VTS closely matched the traditional HTS with an excellent ICC (0.93–0.98) for horizontal and vertical deviations and a good ICC (0.79–0.93) for torsional misalignment. ICC for horizontal and vertical deviations were similar in primary and secondary eye positions, indicating that the VTS performed equally well in different gaze directions. Only in downgaze ICC was somewhat lower (0.79–0.87) for torsional deviations compared to other directions of gaze (0.89–0.93).

For horizontal deviations, the differences between two tests were within the range of ± 6° for interobserver test–retest variability known for the APCT [13, 14]. The traditional HST has a reported uncertainty of up to ±5° [3]. The vertical distribution was narrower than the horizontal (Table 2), indicating that both tests were in closer agreement for vertical deviations with no systematic bias. This was probably due to narrower physiological limits of vertical vergence amplitudes [15].

A slightly higher vergence demand was evident with the VTS test, as horizontal deviations showed on average 2° higher esodeviation than with the HTS. An explanation could be a higher accommodative demand for the VTS compared to the HTS. Although we set the virtual distance at 2.5 m for the VTS, the focal distance of Oculus Go^®^ is in fact 1.3 m. Accommodation in response to this focal distance may induce significant convergence associated with an esotropic shift [5]. A similar esotropic shift was already noted by Yeh et al. [5]. To address this aspect, further investigations are necessary to clarify whether additional plus lenses reduce the esotropic shift with the VTS compared to the HTS.

The 95% LOA of the torsional misalignment between the two tests (Table 2) were in most directions of gaze within the range of the test–retest variability of cyclodeviations measured with the Maddox rod test or synoptophore (4.5°—5°) [16, 17]. The ICC of torsional misalignment was lower than for horizontal and vertical deviations. An explanation could be difficulties of the patients to handle the controller. A reprogramming to a controller with more holding stability such as a computer mouse with a central wheel facilitating fine adjustments may improve the measurement accuracy. The advantage of the standard Oculus Go with the controller was that no powerful computer was needed to use this HMD. Another reason for the larger differences in cyclodeviations between VTS and HTS could be that with the VTS the bar is set back to 0° cyclodeviation for every viewing direction. This probably leads to an overestimation of 0° cyclodeviation with the VST. This is different from the HTS, where the setting of the bar has to be readjusted in every new viewing direction from the already set position. The software of the VTS could be reprogrammed to perform similarly. Differences in torsional alignment, especially in downgaze, could have also been caused by the necessity to reduce the field of view in VTS in some patients. In superior oblique palsy torsional deviation increases much in downgaze, so this could have led to a different height of the torsional deviation.

In recent years, strabismus angles were examined using specially developed video goggles and compared with the Hess screen [12]. On average the authors found good agreement between the two examination methods with a maximum deviation of horizontal squint angles of ≤ 12° and vertical squint angles ≤ 6° [12]. Horizontal values exceeded the usual measurement fluctuations [13, 14]. In another study, ocular deviations were assessed in patients with ocular palsy via a self-constructed Harms digital screen test using an image beamer to display the tangent screen and glasses with red and blue filters for image isolation of each eye [6]. The differences between the HTS and its digital version were similar to our study results showing a mean difference of 1.77° (SD 2.95°) for horizontal deviations and 1.11° (SD 2.23°) for vertical deviations. In the follow-up publication a refined digitized tangent screen test was presented, that allowed definition of the head orientation by an absolute position sensor and measurement of the ocular torsion [7]. The validation results of this test on patients with strabismus have not yet been published. A new eye tracking VR-based ocular objective deviation measurement simulating APCT showed also good agreement between both methods in a cohort of 38 patients [5] (Yeh et al. 2021). ICC of 0.897 for horizontal angles of deviation with 95% LOA of 6.5° was similar to our study results [5]. Miao et al. [4] also found a good agreement of their test with the APCT in 17 patients. Those VR-based tests however do not provide torsional misalignment data.

There are several limitations in our study. VTS was always performed after traditional HTS. This could have led to a higher phoric component due to a longer period of dissociation. Due to the 60° field of view of the Oculus Go, patients with squint angles > 30° could not be tested. Utilization of an HMD with a larger field of view would overcome this limitation. Patients with severe paralytic strabismus were excluded from this study, so comparative data are missing for these patients. They should be collected in a future study as in cases with ocular motility restrictions the software allows manual reduction of the field of view. A further limitation of the study is that it was not possible to change the interpupillary distance (IPD) in the Oculus Go HMD, which could have a prismatic effect on the results. In patients with smaller IPD VR-goggles could act as a base in prism, in case of larger IPD as a base out prism.

## Conclusion

In this study we successfully validated VTS against traditional HTS in patients with strabismus of different origin. The technique is promising but needs further refinement to be implemented in a clinical setting to provide a wider availability of subjective squint angle measurements. With increasing incidence of neurogenic diplopia in the ageing population [18], this would provide ophthalmologists, neurologists and rehabilitation clinics with an easier method to diagnose and follow patients with acquired and often complex strabismus forms.

## Supplementary Information

Below is the link to the electronic supplementary material.

Supplementary file1 (XLSX 34 KB)

## Data Availability

All data generated or analyzed during this study are included in this article. Further enquiries can be directed to the corresponding author.
